# Neuro‐Behçet's Disease Masquerading as Multiple Sclerosis in a Young Male

**DOI:** 10.1002/ccr3.70002

**Published:** 2024-12-15

**Authors:** Sanaz Khodadadi, Reyhaneh Montazeri‐Shatouri, Abdorreza Naser Moghadasi

**Affiliations:** ^1^ Multiple Sclerosis Research Center, Neuroscience Institute Tehran University of Medical Sciences Tehran Iran

**Keywords:** longitudinally extensive transverse myelitis (LETM), misdiagnosis, multiple sclerosis (MS), neuro‐Behçet's disease (NBD)

## Abstract

This case highlights the importance of considering Neuro‐Behçet's disease (NBD) in the differential diagnosis of multiple sclerosis (MS), particularly in patients presenting with neurological manifestations, abnormal magnetic resonance imaging (MRI) findings, and systemic symptoms consistent with Behçet's disease (BD).

## 1 Introduction

Behçet's disease (BD) is a systemic inflammatory vasculitis characterized by recurrent episodes of oral aphthous ulcers, genital ulcers, skin lesions, and ocular involvement. The etiology of BD is unknown. It can also affect the neurological, gastrointestinal, and vascular systems. BD is more prevalent in the Middle East and Far East and affects both genders equally [1].

Neurological involvement occurs in 5%–10% of patients with BD. It is more common in men and is often associated with severe complications. The neurological manifestations of BD are classified into two categories: parenchymal and nonparenchymal disease. Approximately 80% of neurological cases involve parenchymal disease. Parenchymal disease most often affects the brainstem, basal ganglia, or both and is linked to a worse prognosis. Around 10% of cases involve the spinal cord, most often in the cervical and thoracic regions. These lesions can be singular, small, or multifocal. However, they are more often longitudinally extensive, with enhancement that often presents in a ring or diffuse pattern [2]. Nonparenchymal manifestations include arterial vasculitis, dural sinus thrombosis, and aseptic meningitis. Neurological symptoms of BD include extrapyramidal and pyramidal tract disorders, cranial nerve dysfunction, headaches, behavioral changes, and sphincter control issues [3].

Neuro‐Behçet's disease (NBD) is diagnosed clinically, as no specific diagnostic tests are available. It's important to exclude other conditions that mimic NBD before confirming the diagnosis. Magnetic resonance imaging (MRI) is a key tool for differentiating NBD from other conditions. However, when MRI findings resemble those of multiple sclerosis (MS), the diagnostic process becomes more complicated.

We describe a case of NBD that was misdiagnosed as MS because of atypical MRI findings and typical MS symptoms. This case highlights the difficulty in diagnosing NBD and emphasizes the need to include it in the differential diagnosis when patients present with neurological symptoms, unusual MRI findings, and systemic features of BD.

## 2 Case History/Examination

**FIGURE 1:**
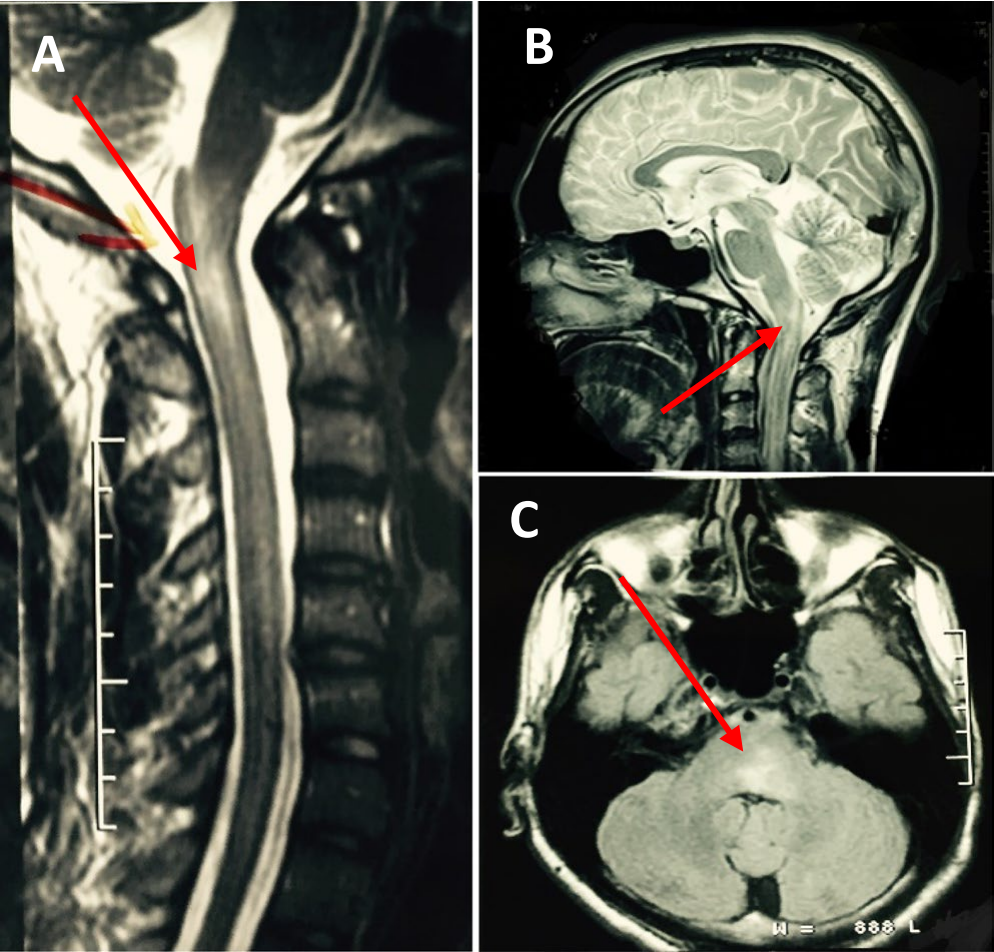
(A) Sagittal T2‐weighted MRI of the cervical spine: The image shows hyperintense lesions within the cervical spinal cord extending from the medulla to C5. (B) Sagittal T1‐weighted MRI: The image demonstrates a hyperintense lesion in the medulla and upper cervical region. (C) Axial T2‐weighted MRI: The image revealed a hyperintense lesion in pons.

A 25‐year‐old Iranian male presented to the emergency department with a one‐year history of gradually worsening weakness and numbness in the lower limbs, along with hypesthesia in the lower limbs and right‐sided perineum. He also reported recurrent oral and genital aphthous ulcers over the past year, with each episode lasting approximately 1 month and healing with scarring. At presentation, the ulcers had persisted for 2 weeks. He also had urinary hesitancy for the past 8 months. Three years before this admission, the patient had experienced neck pain. MRI of the brain and cervical spine (Figure 1) revealed hyperintense lesions in the cervical spinal cord, extending from the medulla to the upper cervical region. His symptoms resolved without any treatment or diagnosis. Six months later, he experienced paresthesia in the breast and axillary regions, along with symmetric erythema, periorbital edema, and diplopia. These symptoms resolved after multiple courses of corticosteroid therapy; however, no diagnostic workup was performed at that time. Later that year, after experiencing recurrent episodes of diplopia, a neurologist diagnosed the patient with MS, and beta‐interferon treatment was initiated. He had no significant medical history and reported using only beta‐interferon for treatment.

The patient was conscious and had normal vital signs. The systemic examination, including cardiopulmonary, chest, and abdominal assessments, was unremarkable. Dermatological evaluation revealed multiple papules on the posterior trunk. Oral examination identified several aphthous ulcers, with none noted in the genital area. Musculoskeletal examination showed swelling and erythema of the right lateral malleolus. Ophthalmic examination was normal, except for a retinal finding of a single cotton wool spot on the temporal side of the right eye. Neurological assessment showed the following: Cranial nerve evaluation revealed normal funduscopic results, intact pupillary light responses, and preserved facial sensory and motor functions. Motor testing revealed normal muscle strength in the upper limbs (5/5), spasticity with reduced strength in the proximal lower extremities (4/5), and normal muscle strength in the distal lower extremities (5/5). The reflex examination revealed symmetrical and normal reflexes in the biceps, brachioradialis, and triceps, with brisk patellar reflexes graded as 3+ and hyperactive ankle reflexes graded as 4+. Sensory testing revealed decreased bilateral pinprick and light touch sensation in the lower limbs, whereas deep sensory testing indicated heightened proprioception in the lower limbs. Cerebellar function was intact, demonstrated by normal performance on finger‐to‐nose and heel‐to‐shin coordination. Gait assessment was impaired, and the Romberg test was positive.

## 3 Methods

**FIGURE 2:**
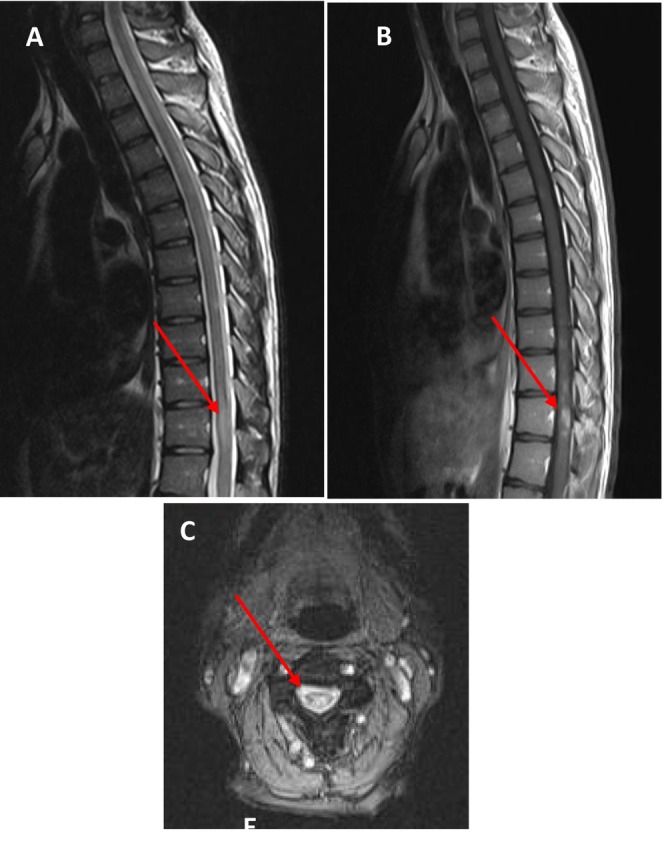
(A) Sagittal T2‐weighted MRI of the thoracic spine without contrast: The image shows a hyperintense lesion. (B) Sagittal T1‐weighted MRI of the thoracic spine with contrast: The image demonstrates an enhanced lesion. (C) Axial T2‐weighted MRI of the spine: The image shows a hyperintense lesion in the central cord of thoracic level.

A brain and spinal MRI showed hyperintense lesions in the thoracic region, with normal findings in the brain and cervical spine (Figure 2). Chest computed tomography (CT) was unremarkable. Laboratory test results are summarized in Table 1. Renal function, liver function, and metabolic panels were normal, except for an elevated white blood cell count (WBC) with increased monocytes. The immunologic panel was negative for perinuclear antineutrophil cytoplasmic antibodies (p‐ANCA), cytoplasmic antineutrophil antibodies (c‐ANCA), fluorescent antinuclear antibody (FANA), anticardiolipin antibodies, antiphospholipid IgG, and Aquaporin 4 antibodies. However, human leukocyte antigen B5 (HLA‐B5) and human leukocyte antigen B51 (HLA‐B51) were positive.

**TABLE 1:** Blood test results.

Test	Result	Reference range
Hematocrit (%)	44.4	41–52
Hemoglobin (g/dL)	15.6	14–17.5
Red blood cells × 10^6^/μL	5.48	4.5–5.5
White blood cells × 10^9^/L	11,040	4–10,000
Neutrophils (%)	56.6	40–60
Lymphocytes (%)	27.6	20–40
Monocytes (%)	14.3	2–8
Platelet count × 10^3^/μL	300	150–400
C‐reactive protein (mg/L)	22.1	< 6
Erythrocyte sedimentation rate (mm/h)	19	< 18
Urea (mg/dL)	20	19–44
Creatinine (mg/dL)	1.07	0.7–1.4
Aspartate aminotransferase (U/L)	10	< 37
Alanine aminotransferase (U/L)	10	< 41
Alkaline phosphatase (U/L)	197	80–306

**FIGURE 3:**
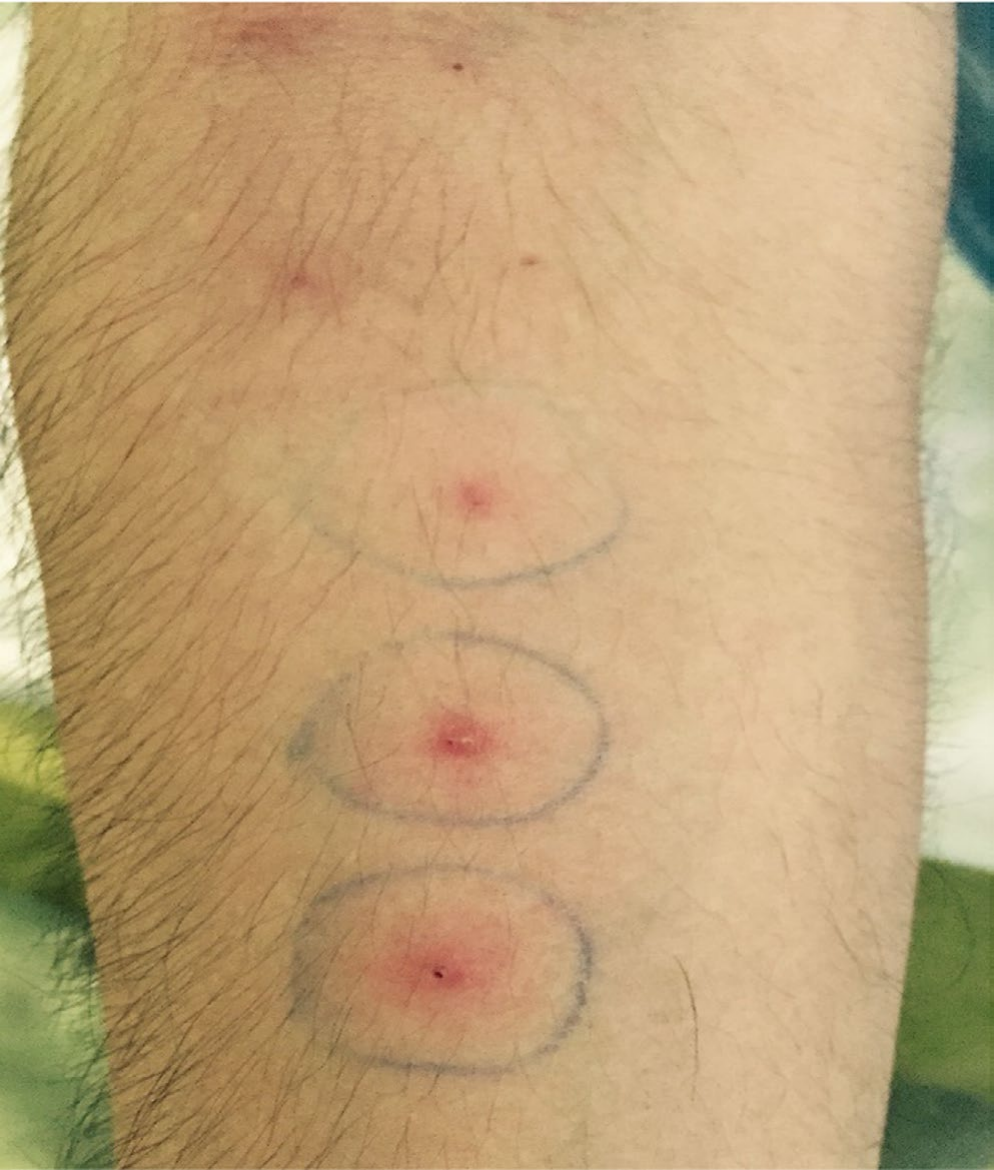
The forearm shows three sites of needle pricks, each marked by a red bump, indicating a positive pathergy reaction.

Serology tests for Wright, Coombs Wright, and rapid plasma reagin (RPR) were negative. Serum immunoglobulins (IgG, IgA, IgM, IgE) and angiotensin‐converting enzyme (ACE) levels were within normal limits. Blood tests for HIV antigen and antibody, hepatitis B surface antigen (HBsAg), hepatitis B surface antibody (Anti‐HBs), hepatitis C antibody (Anti‐HCV), and varicella‐zoster virus (VZV) IgG and IgM antibodies were all negative. Cerebrospinal fluid (CSF) analysis showed a colorless fluid with a glucose level of 45 mg/dL (normal range: 45–75 mg/dL), protein of 50.7 mg/dL (normal range: 15–45 mg/dL), and 20 WBC/μL (10% polymorphonuclear cells, 90% lymphocytes). CSF oligoclonal bands (OCB) were negative. The skin pathergy test was positive (Figure 3). The retinal angiography was performed because of the abnormal finding on the eye examination was normal.

## 4 Conclusion and Results

After excluding other differential diagnoses, including neuromyelitis optica spectrum disorder (NMOSD), sarcoidosis, systemic lupus erythematosus (SLE), and infectious myelitis, a diagnosis of NBD was confirmed. The patient was initiated on monthly intravenous cyclophosphamide (1 g) along with prednisolone (0.5 mg/kg/day) for 14 days, resulting in gradual symptom improvement within 1 week. Six months later, treatment was transitioned to mycophenolate mofetil (500 mg three times daily). At the 24‐month follow‐up, the patient remained stable and well.

## 5 Discussion

NBD is a rare condition that should be considered in the differential diagnosis alongside inflammatory, infectious, or demyelinating disorders of the central nervous system (CNS) [4]. An important differential diagnosis for NBD is MS, as both conditions are prevalent in young adults. However, MS predominantly affects females, whereas NBD is more common in males [5]. Eighty percent of NBD cases present with a relapsing course, and 30% of these patients eventually progress to a chronic disease stage, either immediately or after an initial relapsing phase [2, 6]. NBD can involve different parts of the CNS, including the brainstem (50%), diencephalon (30%), and spinal cord (10%). In a group of 17 patients with NBD presenting with spinal cord involvement, eight had lesions affecting more than half of the spinal cord longitudinally. This suggests that longitudinally extensive transverse myelitis (LETM) is a common inflammatory feature of spinal cord involvement in NBD. The main symptoms of spinal cord involvement, on the basis of previous studies, include hypesthesia, limb weakness, and sphincter or sexual dysfunction. Typically, the symptoms of BD appear before the spinal cord is affected [4]. However, in our case, the patient presented with spinal cord involvement before the onset of systemic symptoms, such as genital and oral aphthous ulcers, which is contrary to previous reports. Ocular involvement is also considered a typical feature of NBD, although specific manifestations are often not well recognized. For instance, diplopia, as seen in our patient, is not common but can occur because of cranial nerve palsy, particularly involving cranial nerves VI and VII [2, 6]. This atypical presentation complicated the diagnostic process and increased the likelihood of misdiagnosis as MS.

Diagnostic tests can help differentiate between MS and NBD. In MS, CSF analysis typically shows positive OCB, with a normal cell count and normal protein levels. In contrast, NBD is characterized by negative OCB, an elevated WBC count primarily consisting of neutrophils that shift to lymphocytes, and mildly elevated CSF protein levels [7]. In previous cases of LETM where CSF analysis was performed, all cases demonstrated elevated protein levels and an increased WBC count [4]. MRI is an important diagnostic tool for assessing spinal cord involvement. In NBD, lesions may be found in the cervical and thoracic regions. These lesions can be singular, small, or multifocal, but they are more commonly longitudinally extensive with enhancement, often presenting in a ring or diffuse pattern [2]. In MS, however, demyelinating plaques are typically located dorsally and laterally, involving less than half of the diameter of the spinal cord on axial imaging [8]. The exact pathophysiology of long spinal cord involvement is unclear. However, evidence suggests that the inflammatory process involves small vessel obstruction along the small veins on the surface of the spinal cord, leading to congestion and edema of the central gray matter, as indicated by hyperintensity on imaging [9, 10].

Immunosuppressive treatment combined with corticosteroids is recommended for severe forms of NBD, such as cerebellar deep venous thrombosis, myelopathy, and meningoencephalitis. Although the majority of cases are treated with azathioprine and intravenous methylprednisolone [11], limited studies have demonstrated effectiveness with cyclophosphamide [12], as seen in our patient. On the basis of previous studies, the treatment outcome for patients with LETM is generally worse compared to those with localized lesions [4, 8, 13]. However, after a 2‐year follow‐up, our patient remained well without any neurological sequelae.

NBD is a rare condition that requires a diagnosis of exclusion, especially to differentiate it from other conditions with similar symptoms, such as MS. Our case highlights the importance of including NBD in the differential diagnosis for patients presenting with neurological symptoms, LETM findings on MRI, and systemic features consistent with BD. Early identification of NBD is essential, as spinal involvement can be disabling, and timely treatment can prevent further progression.

## Author Contributions

**Sanaz Khodadadi:** data curation, writing – original draft. **Reyhaneh Montazeri‐Shatouri:** data curation, writing – original draft. **Abdorreza Naser Moghadasi:** conceptualization, supervision, writing – review and editing.

## Consent

Written informed consent has been obtained from the patient to publish this report by the journal's patient consent policy.

## Conflicts of Interest

The authors declare no conflicts of interest.

## Data Availability

The data are available from the corresponding author upon reasonable request.
